# Cryogenic Differential Amplifier for NMR Applications

**DOI:** 10.1007/s10909-018-02130-1

**Published:** 2018-12-17

**Authors:** V. V. Zavjalov, A. M. Savin, P. J. Hakonen

**Affiliations:** Low Temperature Laboratory, Department of Applied Physics, Aalto University, PO Box 15100, 00076 Aalto, Finland

**Keywords:** Cryogenic amplifier, HEMT, Low-noise, Differential input

## Abstract

We have designed and characterized a cryogenic amplifier for use in $^3$He NMR spectrometry. The amplifier, with a power consumption of $\sim 2.5$ mW, works at temperatures down to 4 K. It has a high-impedance input for measuring a signal from NMR resonant circuit, and a 50 ${\Omega }$ differential input which can be used for pick-up compensation and gain calibration. At 4.2 K, the amplifier has a voltage gain of 45, output resistance 146 ${\Omega }$ and a 4.4 MHz bandwidth starting from DC. At 1 MHz, the voltage and current noise amount to 1.3  $\text{ nV }/\sqrt{\text{ Hz }}$ and 12  $\text{ fA }/\sqrt{\text{ Hz }}$, respectively, which yields an optimal source impedance of $\sim 100$ k${\Omega }$.

## Introduction

Many scientific and research applications benefit from the use of cryogenically cooled amplifiers. Reduction in the amplifier operation temperature leads to significant improvement of the signal-to-noise ratio, which finally improves sensitivity of measurements.

Nowadays, almost all commercially available transistors and operational amplifiers use silicon technology, and they do not work below 10 K. On the other hand, most cryostats have a 4 K plate or liquid helium bath where cold amplifiers can be placed. With silicon-based amplifiers, overheating with temperature stabilization can be used [1] for normal operation. Many non-silicon transistors used previously for cold amplifiers [2] are not available anymore. Now there are a few types of low-noise non-silicon transistors produced commercially which are designed for GHz frequencies. They include GaAs HEMT-technology FET transistors made by Avago (ATF series), CEL (CE series) or TriQuint (TGF series) and SiGe:C npn bipolar transistors made by Infineon (BFP series). These transistors can be used not only at high frequencies [3–5] but also for low frequency applications [6–14]. The performance of cooled low-frequency amplifiers has also been promoted by special, foundry-made transistors [15].

**Fig. 1:**
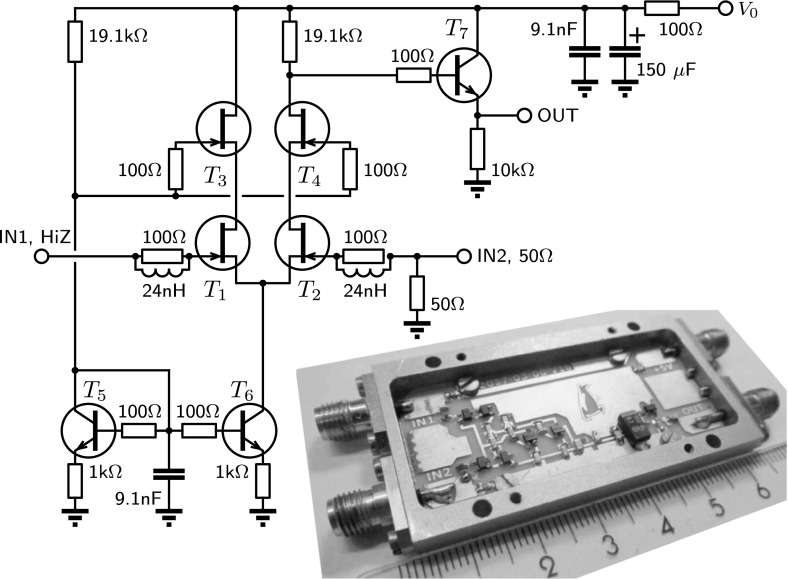
Schematics and a photograph of component layout (Color figure online)

In this paper, we present a cryogenic amplifier based on easily-available, commercial Avago ATF-33143 HEMT components and Infineon BFP640 npn transistors. The goal has been to design a preamplifier for NMR experiments which operate around 1 MHz and utilize tank circuits with high impedance ($\sim 100$ k${\Omega }$).

## Design

The schematics and a photograph of the amplifier are shown in Fig. 1. The differential cascode input stage consists of four HEMT transistors $T_1-T_4$. The stage is powered by a current supply made of two npn transistors $T_5-T_6$. The current is set by the bias voltage $V_0$ and a 19.1 k${\Omega }$ resistor (Note that base–emitter voltage of $T_5$ and $T_6$ is close to 0.75 V). Cascode design together with non-symmetric inputs are used to reduce the Miller effect [16]. This effect takes place when the output voltage couples to the input through transistor’s parasitic capacitance, and then, after amplification, decreases the total gain. A 50 ${\Omega }$ resistor on input 2 forms a divider with the parasitic capacitance which reduces the effect. In equilibrium, the current in both arms is half of the total current, and the output of the amplifier is biased near $V_0/2$. A follower with npn transistor $T_7$ is used to reduce the output resistance of the amplifier. All transistors have 100 ${\Omega }$ resistors connected to their bases/gates. These resistors are needed to damp high-frequency resonances caused by parasitic inductances of the circuit and to improve the stability of the amplifier. At the input terminals, the 100 ${\Omega }$ resistors are shunted by 24 nH inductors at the relevant signal frequencies. The bias voltage line is connected through an RC filter with a 100 ${\Omega }$ resistor, a 150 $\upmu$F tantalum capacitor and a 9.1 nF ceramic capacitor.

For the input stage, we are using Avago ATF-33143 HEMTs. We have tested transistors with smaller gate areas (ATF-34143, ATF-35143) and obtained similar results. For the current supply and the output follower, we use Infineon BFP640 npn SiGe transistors. Passive components for cryogenic applications need to demonstrate both mechanical and electrical stabilities at low temperatures. We are using ceramic C0G capacitors [17], tantalum capacitors [18], and metal-film (thin-film) resistors.

With the bias voltage $V_0=4$ V, the current source gives $\sim 0.2$ mA and the total power consumption of the device amounts to $\sim 2.5$ mW. Low power consumption has certain disadvantages, namely high output resistance and low slew rate. This makes the amplifier performance to be dependent on the output circuit. If the amplifier is connected to an output line with capacitance, two effects are observed at high frequencies: the gain drops because the line capacitance forms an RC-filter with the output resistance; at high amplitudes, the signal is distorted because the output current of the amplifier is limited and this restricts the charging rate of the line capacitance. The second effect can be estimated using the known parameters of the amplifier: the current limit is determined by the bias voltage and the resistor in the output emitter follower, $I_{\text{ max }} \sim 0.4$ mA. The charging rate is constrained by $\dot{V} = 2\pi f\,V = I_{\text{ max }} / C_{\text{ line }}$. For $C_{\text{ line }}=500$ pF and at $f=1$ MHz, the distortion starts at an output amplitude of $V\sim 130$ mV. This estimation agrees with our measurements. Using shorter output cables or adding an additional, more powerful amplifier on the upper part of the cryostat would improve the amplifier performance.

## Measurements

**Fig. 2:**
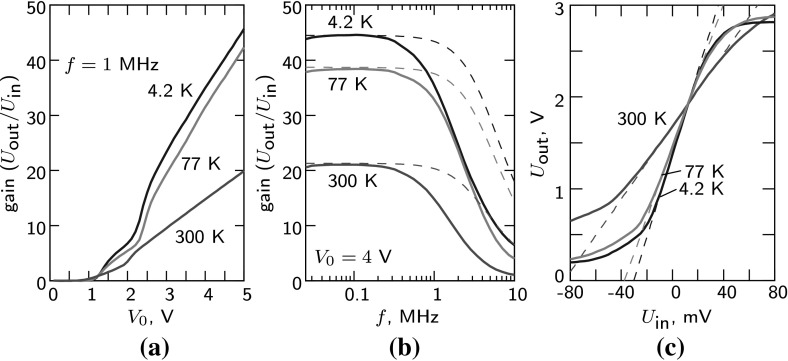
**a** Measured AC gain at 1 MHz as a function of the bias voltage $V_0$. **b** Measured AC gain at $V_0 = 4$ V as a function of frequency. The 1 MHz cutoff frequency here is determined by the output resistance of the amplifier ($\sim 150\, {\Omega }$) and the capacitance of output cables ($\sim 580\, \hbox {pF}$). The obtained intrinsic bandwidth $\sim 4$ MHz is shown by dashed lines. **c** DC response of the amplifier ($U_{\text{ out }}/U_{\text{ in }}$). Measurements have been done at $T=300$ K, 77 K and 4.2 K (Color figure online)

To obtain output parameters of the amplifier (gain, bandwidth and the output resistance), we measured the gain as a function of frequency for a few different configurations: amplifier connected directly to a measurement device (lock-in amplifier); 1 nF capacitor added between the amplifier and the lock-in; a 60 ${\Omega }$ resistor to the ground added after the capacitor. All the data were fitted simultaneously with a simple electrical model with four parameters: gain, bandwidth, the output resistance and the cable capacitance. Measurements were done at room temperature (300 K), temperature of liquid nitrogen (77 K) and liquid helium (4.2 K). Typical cable capacitance in our setup was $\sim 580$ pF and temperature independent. Other parameters are presented in Table 1. In all measurements presented here, we used input 1 while input 2 was shunted to ground by 50 ${\Omega }$ resistor. With swapped input connections, we obtained very similar gain characteristics as expected for a differential amplifier. The measured gain of the amplifier (without the additional capacitor and grounding resistor on the output) is plotted as a function of bias voltage $V_0$ (at 1 MHz) and frequency (at $V_0=4$ V) in Fig. 2a, b, respectively. The measured cutoff frequency $\sim 1$ MHz in Fig. 2b was determined mostly by capacitance of the output cable which formed an RC filter with the output resistance of the amplifier. The obtained intrinsic gain of the amplifier extends over DC$\ldots$4 MHz, and it is illustrated in Fig. 2b by dashed curves. In Fig. 2c, we present a DC response of the amplifier ($U_{\text{ out }}/U_{\text{ in }}$). In Table 1, we provide 1 dB compression ranges obtained from this measurement. They show input voltage ranges where the response deviates from linear function by less then 1 dB (in power units).

**Table 1:** Characteristics of the amplifier measured at three different temperatures

Temperature *T*, K	300	77	4.2
Gain $K_0$ ($U_{\text{ out }}$/$U_{\text{ in }}$)	21.3	38.7	44.5
$R_{\text{ out }}$, ${\Omega }$	233	128	146
Cutoff frequency $f_\mathrm{c}$, MHz	3.23	4.02	4.41
1 dB compression range, mV	$-20.6$–35.6	$-7.93$–14.4	$-5.13$–13.2
Voltage noise, nV/$\sqrt{\text{ Hz }}$			
At 10 kHz	35.2	14.9	6.0
At 100 kHz	8.4	3.6	2.1
At 1 MHz	3.8	1.6	1.3
Current noise, fA/$\sqrt{\text{ Hz }}$			
At 10 kHz	17.5	6.2	6.4
At 100 kHz	30.9	9.3	7.7
At 1 MHz	83.3	21.4	14.5
Optimal source impedance, ${\Omega }$			
At 10 kHz	2.0M	2.4M	930k
At 100 kHz	270k	390k	270k
At 1 MHz	45k	74k	89k

Bias voltage $V_0=4$ V has been employed in all measurements. Current noise at all frequencies was measured with resistive source. More accurate measurement with a resonant circuit gave 11.8 fA/$\sqrt{\text{ Hz }}$ at 1 MHz

**Fig. 3:**
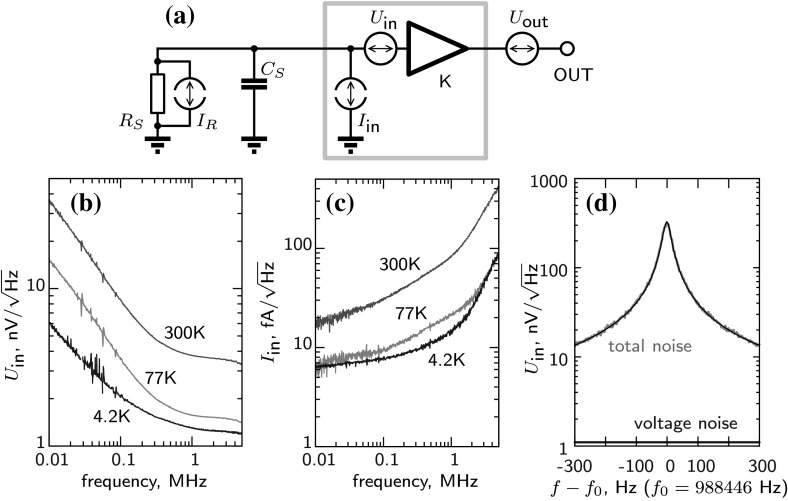
Model for noise measurements (**a**), measured voltage (**b**) and current (**c**) noise of the amplifier. Measurements have been done at 300 K, 77 K and 4.2 K using bias $V_0=4$ V. **d** Noise measured using a high-*Q* tank circuit at the input of the amplifier. The curve is a sum of small input voltage noise 1.3 nV/$\sqrt{\text{ Hz }}$ and input current noise 11.8 fA/$\sqrt{\text{ Hz }}$ imposed on the impedance of the tank circuit (Color figure online)

A model used for our noise measurement analysis is shown in Fig. 3a. The amplifier consists of a noiseless amplifier with voltage gain *K*, input voltage noise source $U_{\text{ in }}$, and input current noise source $I_{\text{ in }}$. The input of the amplifier is connected to a resistor $R_\mathrm{S}$ which also works as a source of thermal noise $I_\mathrm{R}^2 = 4k_BT/R$, where *T* is the temperature of the resistor. Capacitance $C_\mathrm{S}$ of the input circuit is also taken into account. Output of the amplifier is connected to a digital oscilloscope which is used to record noise spectra. The noise source $U_{\text{ out }}$ represents the noise of the oscilloscope.

If the noise sources are not correlated, we can make a square sum of average noise voltages produced by each source. Then the square of the measured noise spectral density becomes

1 $$\begin{aligned} U^2= & {} U_{\text{ out }}^2 + K^2 \left[ U_{\text{ in }}^2 + \left( I_{\text{ in }}^2 + I_\mathrm{R}^2\right) |Z_\mathrm{S}|^2 \right] ,\nonumber \\ |Z_\mathrm{S}|^2= & {} \frac{R_\mathrm{S}^2}{1+(\omega R_\mathrm{S} C_\mathrm{S})^2}. \end{aligned}$$

here $Z_\mathrm{S}$ is the total impedance of the source ($R_\mathrm{S}$ and $C_\mathrm{S}$ in parallel) and ${\omega }$ denotes the angular frequency.

It is easy to find $U_{\text{ out }}$ and $U_{\text{ in }}$ by measuring two noise spectra: one with the amplifier powered off ($K=0$) and another with shorted input ($R_\mathrm{S}=0$). For finding $U_{\text{ in }}$, one has to subtract one spectrum from another and divide square root of the result by known gain.

To determine the input current noise $I_{\text{ in }}$, we recorded noise spectra with nonzero source resistance $R_\mathrm{S}$. The noise with $R_\mathrm{S}=0$ was subtracted (removing the effects of $U_{\text{ out }}$ and $U_{\text{ in }}$), and the result was converted to amplifier input. This gives us a source-dependent component of the input noise, $(I_{\text{ in }}^2 + I_\mathrm{R}^2) |Z_\mathrm{S}|^2$. For small $R_\mathrm{S}$, the main contribution is thermal noise. A cutoff frequency $1/R_\mathrm{S} C_\mathrm{S}$ is clearly seen on the corresponding noise spectra, which allowed us to obtain an estimate for the capacitance at the input $C_\mathrm{S}=6.7$ pF. The input capacitance of the transistor itself is near 1 pF, the rest is determined mostly by SMA connector and the PCB design. Knowing $C_\mathrm{S}$, we evaluated the impedance $Z_\mathrm{S}(\omega )$ and extracted the current noise. We did this using $R_\mathrm{S}=10$ M${\Omega }$ at which the thermal current noise $I_\mathrm{R}$ is smaller. The obtained values for $U_{\text{ in }}$ and $I_{\text{ in }}$ are displayed in Fig. 3b, c.

The voltage noise determination described above was well defined and straightforward, but the measurement of the current noise was more tricky: a large thermal noise part had to be subtracted from the result. To decrease the thermal noise $I_\mathrm{R}$, one has to use huge source resistances, but this limits the bandwidth because of the *RC*-filtering by the input capacitance of the amplifier. In addition, the parasitic input resistance may affect the result if it is comparable with the source resistance.

In order to improve the accuracy of the current–noise measurements, we have made another experiment utilizing a resonant circuit as a high-impedance source, similarly to the work in Ref. [6]. The circuit was formed by a niobium coil with $L=100$ $\upmu$H and a capacitor with $C=200$ pF to obtain a resonance frequency near 1 MHz. We used an American Technical Ceramics porcelain capacitor (100B series) which has very low dissipation. Any resistive metal near the coil leads to extra dissipation as well as thermal noise. To avoid this, we used a superconducting wire without normal-metal matrix. The circuit was placed in a solder-covered copper box which worked as a superconducting enclosure. We concluded that if a small piece of normal metal inside the enclose does not decrease the *Q*-value below a few thousand then it has no substantial effect on the noise. This confirms that the measured noise is originated from the amplifier.

The output voltage noise was recorded as described above. The gain of the amplifier was calibrated using an external noise source connected to input 2. Near 1 MHz, the equivalent input noise contains a constant voltage noise ($\approx 1.3$ nV/$\sqrt{\text{ Hz }}$) and a Lorentzian-shaped noise peak, produced by current noise imposed on the impedance of the tank circuit.

With all the precautions, we managed to reach $Q\approx 1.2\cdot 10^6$, which corresponds to the maximum impedance $Z=0.9$ G${\Omega }$ and an effective dissipative resistance 0.5 m${\Omega }$ in series with the coil. This confirms that the input impedance of the amplifier is higher than 0.9 G${\Omega }$. It is hard to make good noise measurements with such a high *Q*-value circuit because of the long time needed for good frequency resolution and frequency drifts during this time. For avoiding these problems in our tank circuit noise measurements, we added a small resistor to reduce the *Q*-value to $40\cdot 10^3$ ($Z=28$ M${\Omega }$). The noise curve measured with such a circuit is depicted in Fig. 3d. According to the obtained data, the current noise of the amplifier is 11.8 fA/$\sqrt{\text{ Hz }}$, which is in a good agreement with the above-described measurements with a 10 M${\Omega }$ resistive source.

We made and tested a few similar amplifiers. Altogether, it was easy to make a working device and to get the expected characteristics. The gain is determined by the input HEMT transistors, the characteristics of which may vary. We observed about 15% scatter in the gain value between different devices. The balancing of the transistors is also important: one has to check that the DC response is symmetric and replace HEMT transistors if needed. We did not observe any noticeable change in the amplifier characteristics after 10–20 fast cooling and heating cycles. Floating of input 1 can cause electrostatic damage to the input HEMT transistor. To avoid this one can ground input 1 by a 1 M${\Omega }$ resistor, but depending on the impedance of the signal source, the noise contribution of this resistor can be significant. Noise coming from input 2 can increase both the current and voltage noise of the amplifier. One has to incorporate a sufficient amount attenuation at low *T* when connecting input 2 to room temperature devices.

**Fig. 4:**
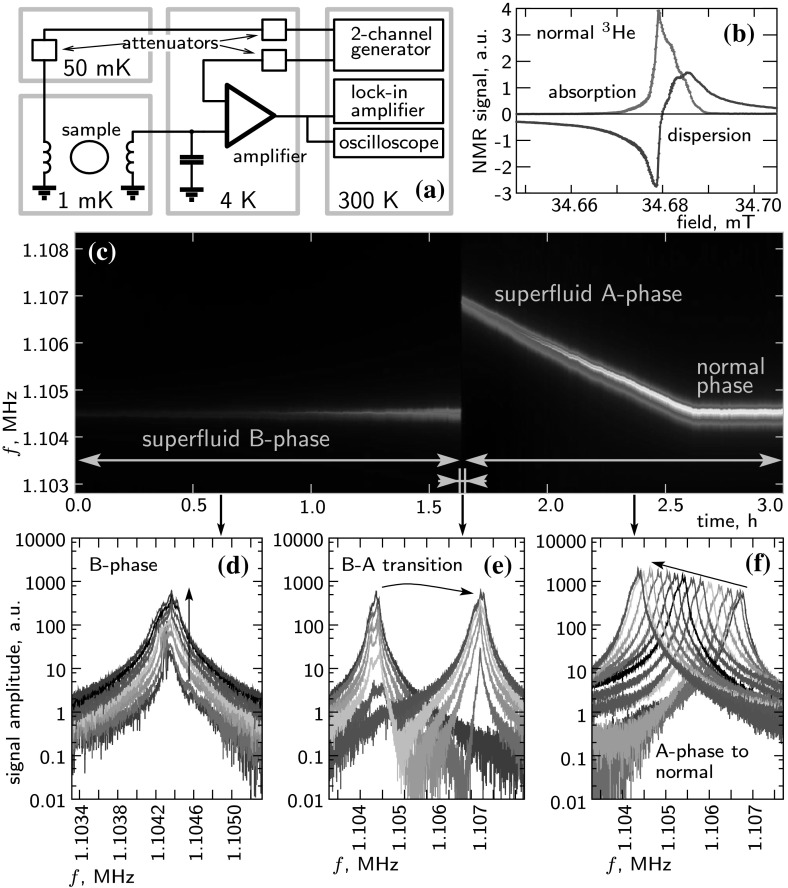
Application example. **a** Measurement setup (see text). **b** Typical continuous-wave NMR signal in normal $^3$He at pressure 29.5 bar, temperature $T=20$ mK and frequency $f=1.10$ MHz. Base level of the signal is near zero because of the compensation imposed to the second input of the amplifier. **c** Pulsed NMR measurement during warming up at the same pressure and NMR frequency. Two phase transitions can be seen when $^3$He traverses through B and A superfluid phases and warms up to the normal phase. Each vertical slice of the picture is a power spectrum of a signal recorded by the oscilloscope after a short excitation pulse from the generator. Examples of such spectra are shown below: **d** in the B-phase, **e** during B to A transition, and **f** in A and normal phases. Black arrows on the plots show evolution of the signal during warming up (Color figure online)

## Application example

We have used the amplifier for NMR experiments in superfluid $^3$He. Schematics of the NMR spectrometer is shown in Fig. 4a. The $^3$He sample is located in a magnetic field of about 35 mT. A pair of crossed coils is used to apply a radio-frequency excitation from a generator and to pick up a signal from precessing magnetization. The pick-up coil ($L\sim 50\,\upmu$H) together with a capacitor forms a tank circuit with a resonant frequency 1 MHz, $Q \sim 200$ and an on-resonance resistance of $\sim 70$ k${\Omega }$. This source resistance is close to the optimal impedance of the amplifier, which means that effects of voltage and current noise are comparable. Using the values from Table 1, one obtains for the total input noise 2.3 nV/$\sqrt{\text{ Hz }}$. Even outside the nuclear magnetic resonance condition, there is a coupled signal on the amplifier due to the mutual inductance and capacitance between the excitation and pick-up coils. We are using the second input of the amplifier to compensate for this signal. This compensation is not affected by possible drifts of the amplifier gain and can be used also for calibration of the NMR signal amplitude. Output of the amplifier is connected to a lock-in amplifier and to an oscilloscope.

In Fig. 4b, a typical continuous-wave NMR signal in normal $^3$He is shown to provide a qualitative understanding of signal-to-noise ratio in our measurements. Figure 4c–f demonstrates another type of measurement, pulsed NMR. A short excitation pulse is used to excite precession of magnetization, and free-induction decay is recorded by oscilloscope. In Fig. 4c, spectra of such signals are presented as a color density plot with time and frequency coordinates. The measurement has been done during warming up from superfluid B-phase of $^3$He, through superfluid A-phase to the normal phase. Both transitions and all the usual features such as change of magnetic susceptibility and temperature-dependent frequency shift in the A-phase are seen. In Fig. 4d–f, individual spectra (slices of the color plot) are shown.

## Conclusions

A differential cryogenic amplifier has been developed and used for high-resolution NMR measurements. The amplifier consumes only 2.5 mW of power, which facilitates installation of many such amplifiers on a 4 K stage of a regular dilution refrigerator. The input current noise of the amplifier is 12 fA/$\sqrt{\text{ Hz }}$ at 1 MHz, which renders our amplifier excellent for high-*Q*, tuned NMR probes up to impedances of a few hundred k${\Omega }$. For the intended NMR applications, it is also important to be able to compensate for the cross-talk input signal on the first amplifier stage and to measure the amplifier gain in situ using the differential input. Our design can be operated even at a smaller power consumption level, but with a slight loss in the amplifier gain. In the future, after optimization for lower bias voltage operation, we plan to position the amplifier on the still plate of a dilution refrigerator, which would further help in minimizing possible external interferences and parasitic impedance problems in cryogenic input circuitry.
